# Supplementary material for: Sex-specific nonlinear DNA methylation aging trajectories reveal biomarkers of cancer risk and inflammation
Source: Genome Biol. 2026 Feb 4;27:2. doi: 10.1186/s13059-026-03952-z (PMC12870970; doi:10.1186/s13059-026-03952-z)
Supplement: Supplementary file 1 — Additional file 1. Supplementary Figures S1-13. [file 13059_2026_3952_MOESM1_ESM.pdf]

Supplementary Figures:

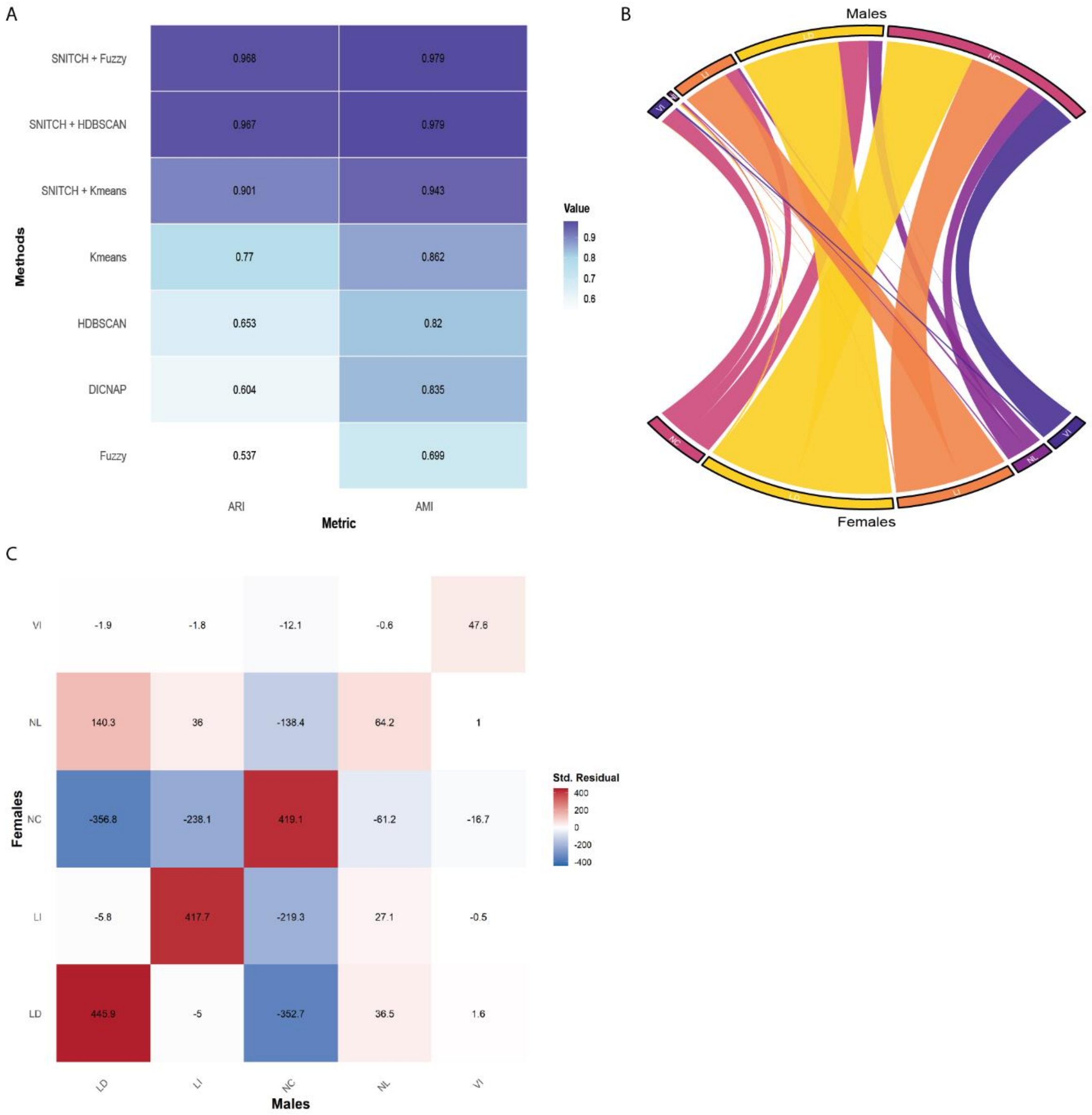

Fig. S1: **A**, Benchmark of SNITCH compared to stand-alone unsupervised clustering methods and DICNAP. **B**, Conserved CpGs between male and female clusters. **C,** Chi-square test for the conservation of CpGs among female and male clusters.

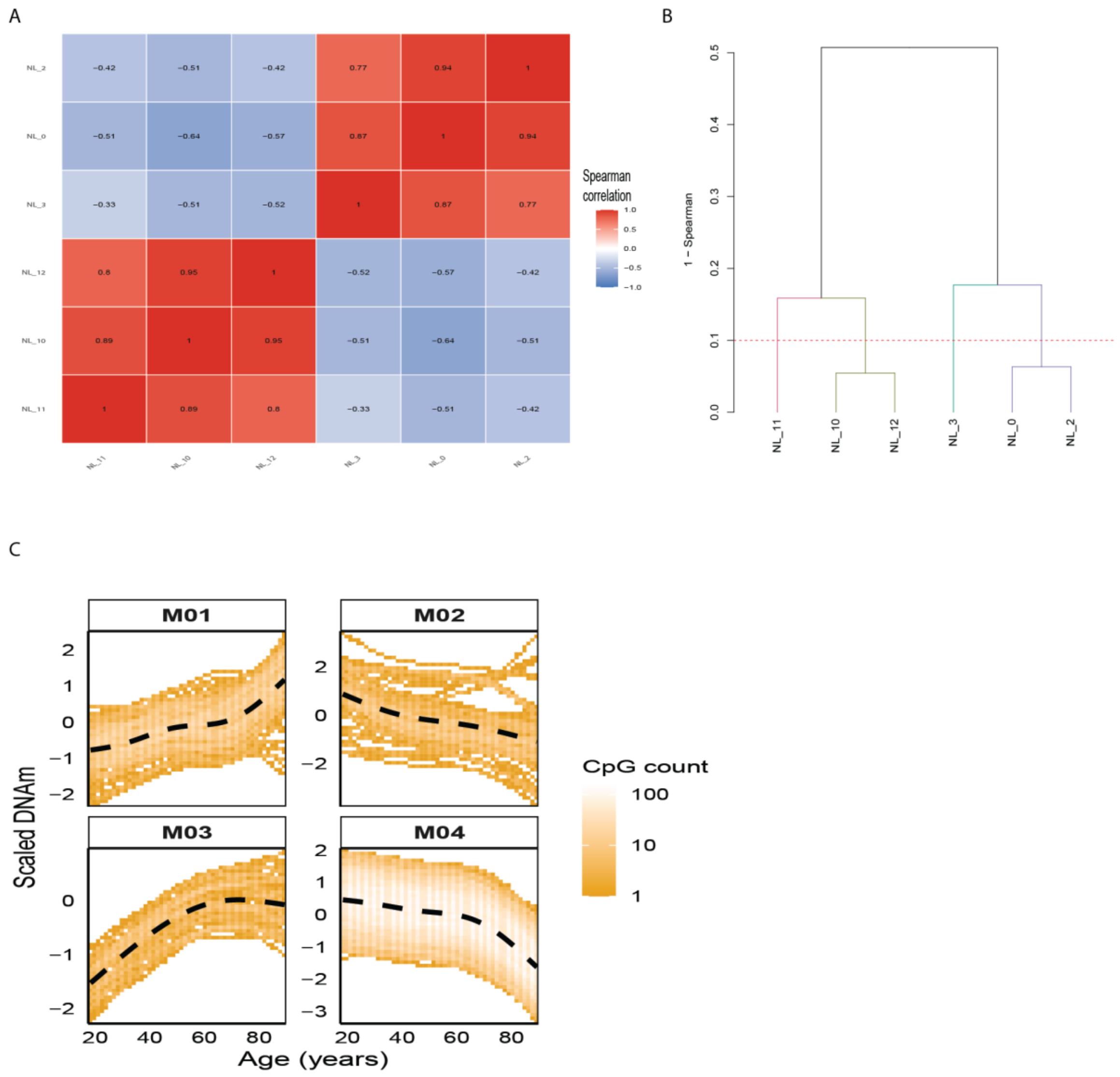

Fig. S2: **A**, Correlation matrix between the NL clusters eigenvalues in females. **B,** Dendrogram representing the similarity cut-off for cluster merging in females. **C,** Resulting cluster after merging in females. Beta values were centered and scaled prior to FPCA and unsupervised

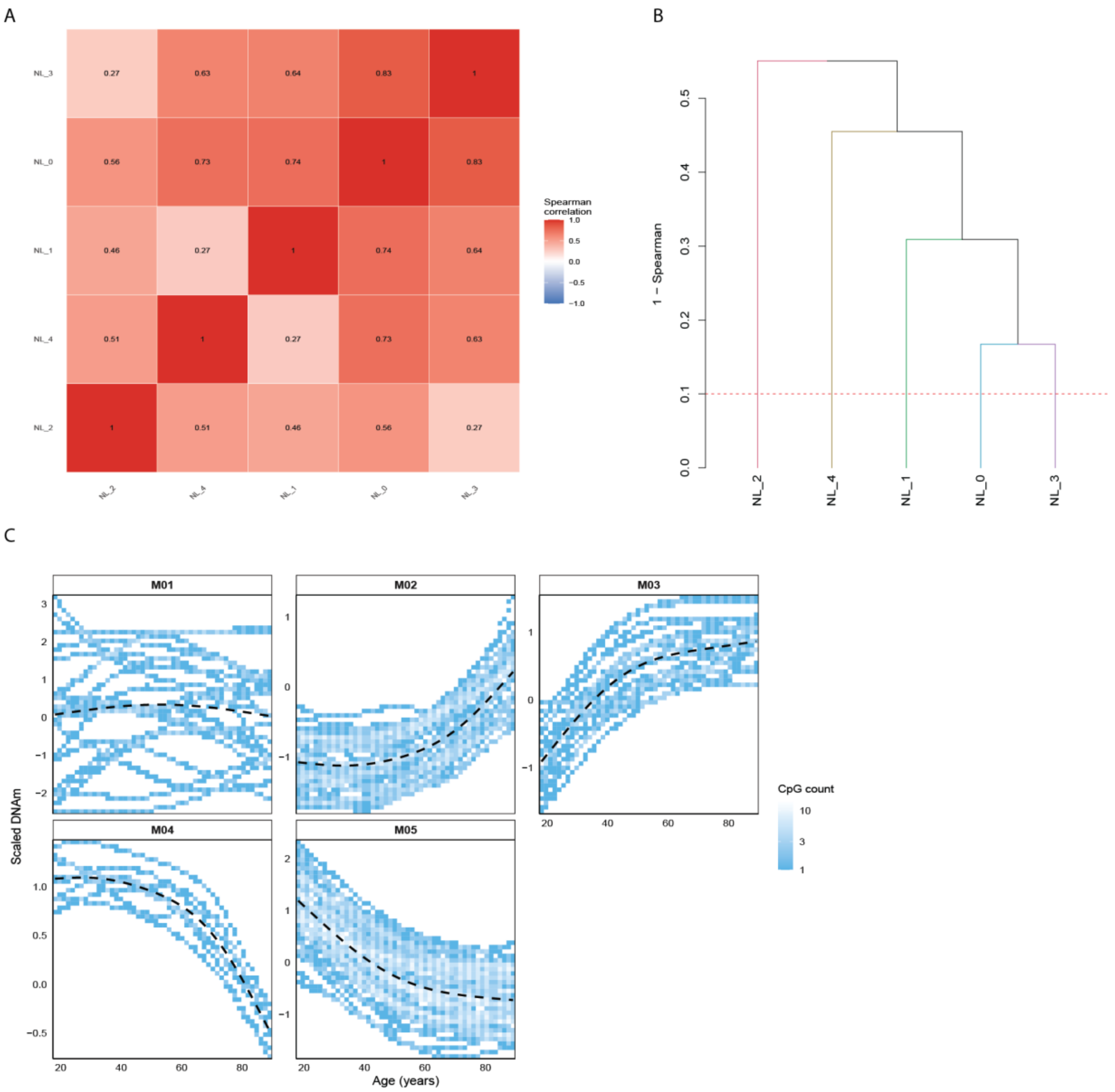

Fig. S3: **A**, Correlation matrix between the NL clusters eigenvalues in males. **B,** Dendrogram representing the similarity cut-off for cluster merging in males. **C,** Resulting cluster after merging in males. Beta values were centered and scaled prior to FPCA and unsupervised

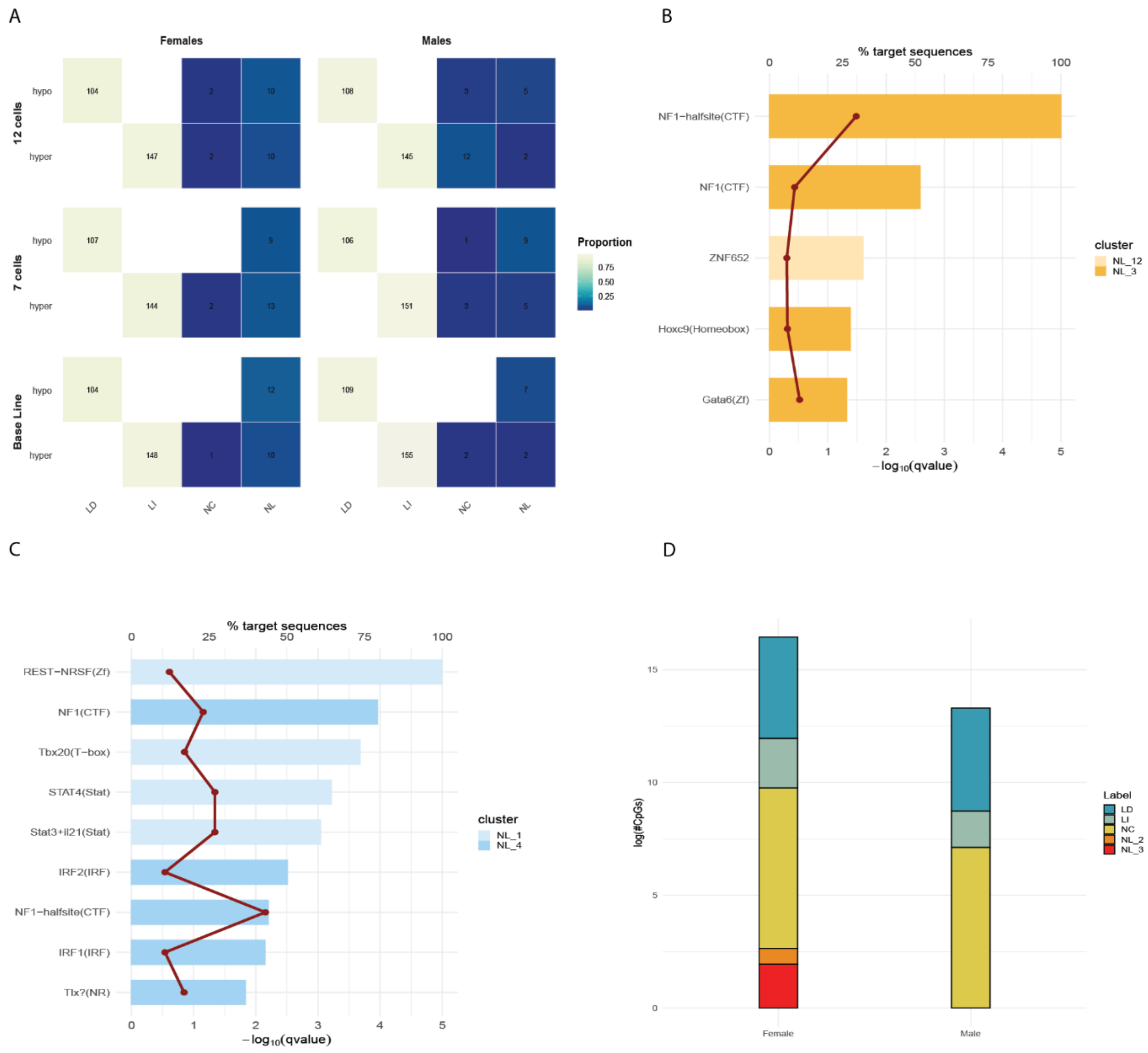

Fig. S4: **A**, Enrichment of CpG labels across age-CpGs identified in the EWAS study by Roy R. et al.. **B**, Cluster-wise motif enrichment analysis among females NL CpGs. **C**, Cluster-wise motif enrichment analysis among males NL CpGs. **D**, Distribution of the CpGs used to estimate C-Reactive Protein levels among males and females aging classification.

A

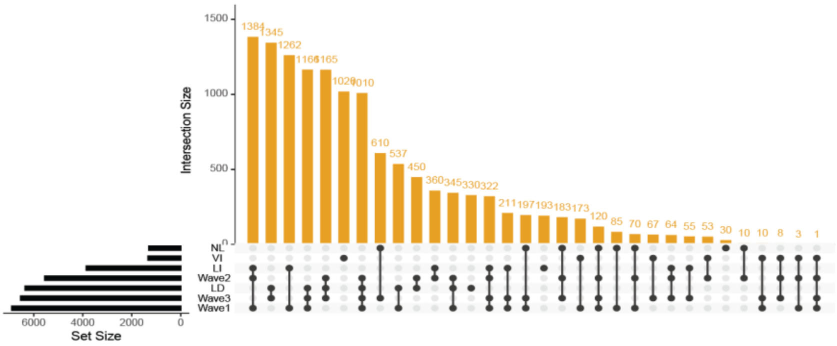

B

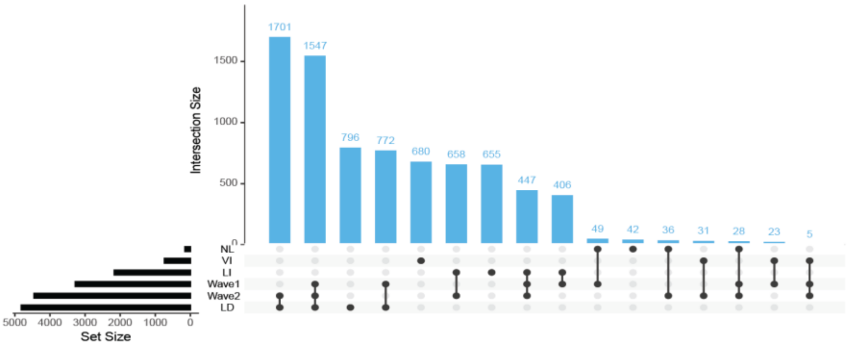

C

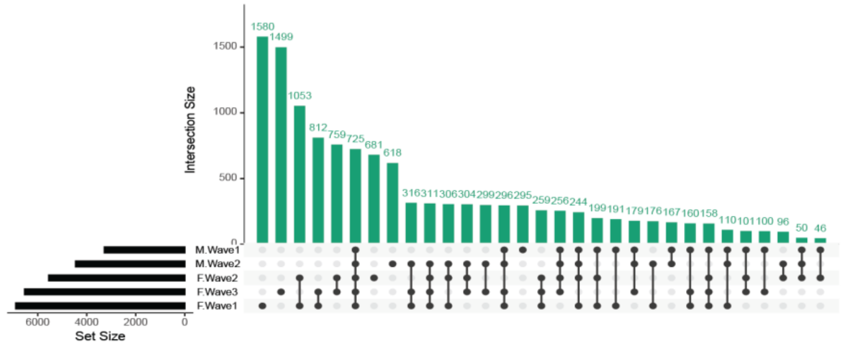

Fig. S5: The overlapping CpGs between waves of regulations and NL clusters in females (**A**), males (**B**), and between male and female waves (**C**).

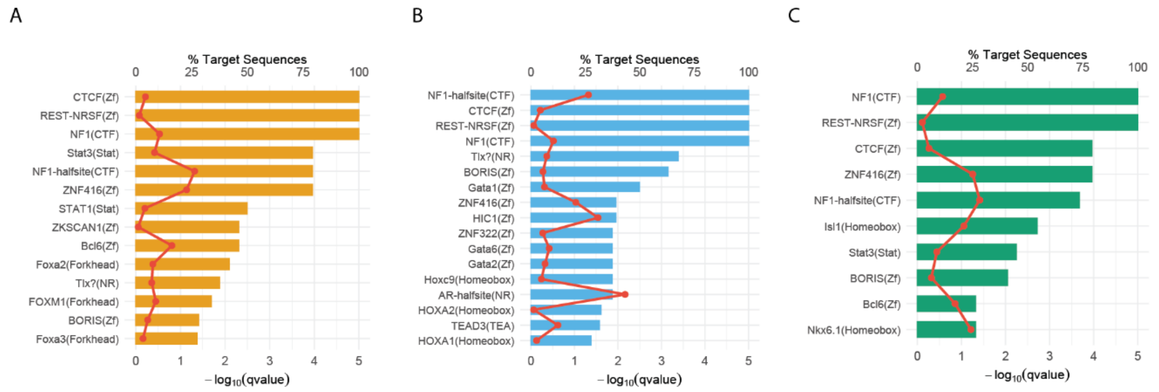

Fig. S6: Motif enrichment analysis among common CpGs identified across waves of dysregulation in females (**A**), males (**B**), and across sex (**C**).

A

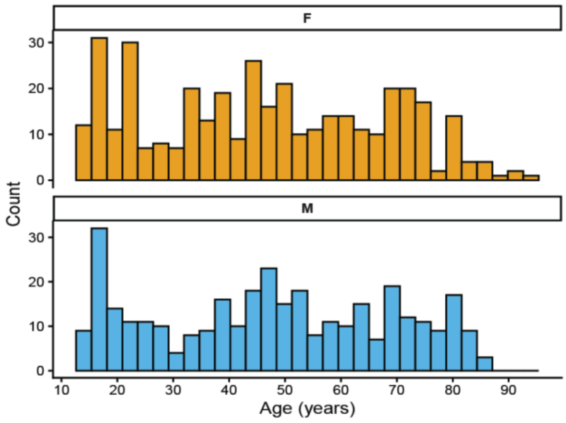

B

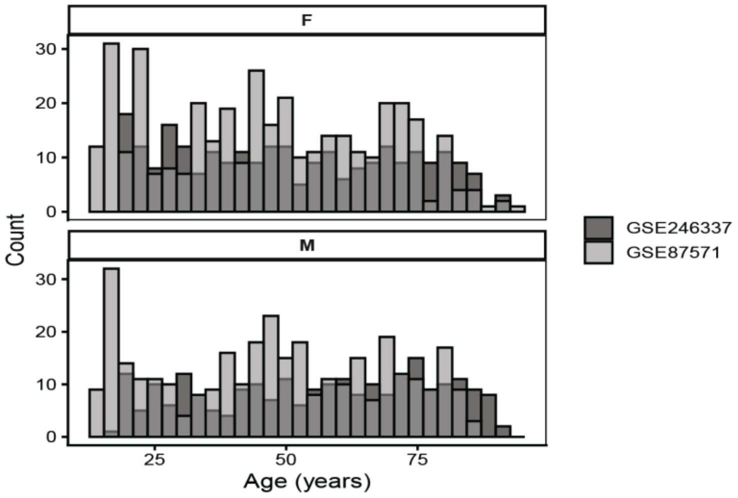

Fig. S7: **A,** Age distribution in the GSE87571 cohort in males and females. **B,** Comparison of the age distribution amongst the two cohorts in males and females (GSE246337 in dark grey, GSE87571 in light grey).

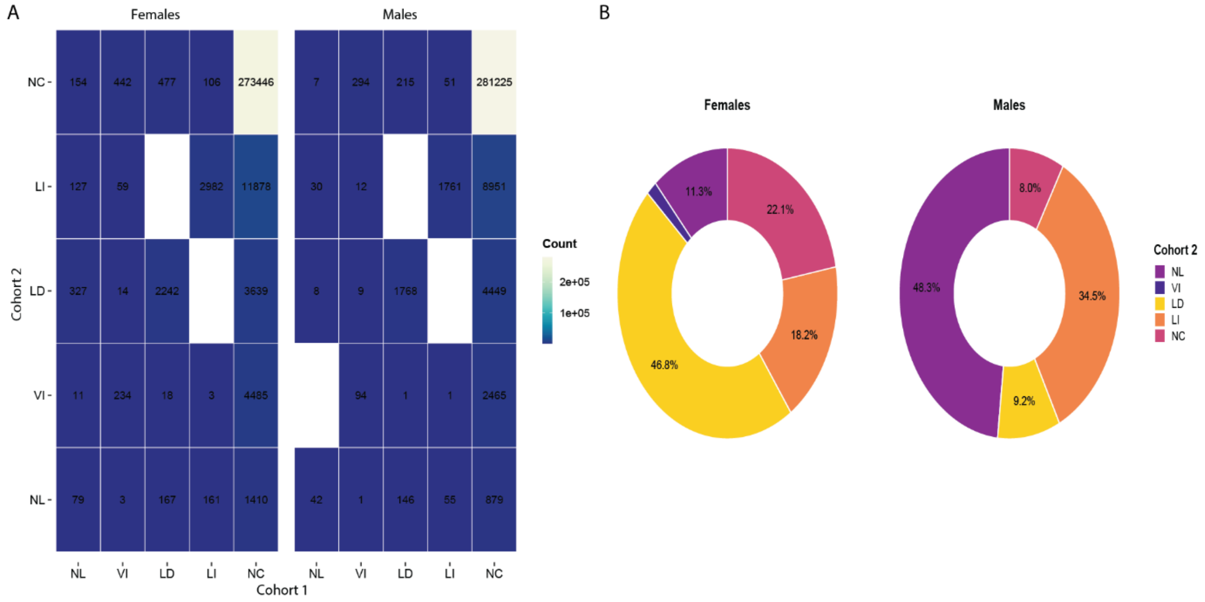

Fig. S8: **A,** Overlap of the classification of CpGs between the two cohorts and across sexes. **B,** Repartition of the classification of NL CpGs in Cohort 1 among the labels assigned in Cohort 2. Cohort 1: GSE246337: Cohort 2: GSE87571.

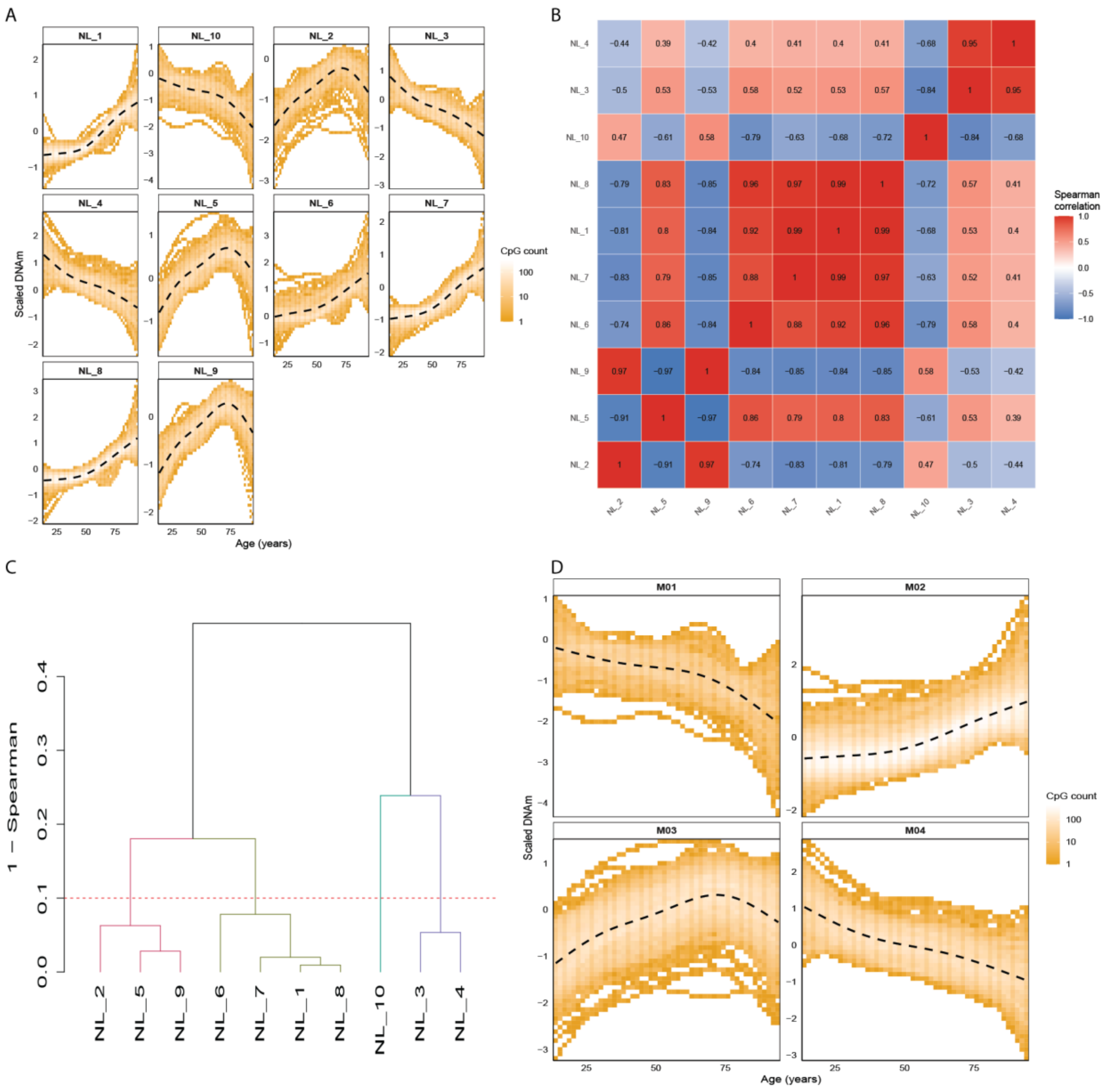

Fig. S9: **A**, Non-linear clusters identified in females after the SNITCH pipeline in Cohort 2. **B,** Correlation matrix between these NL clusters' eigenvalues. **C,** Dendrogram representing the similarity cut-off for merging those clusters. **D,** Resulting cluster after merging in females. Beta values were centered and scaled prior to FPCA and unsupervised.

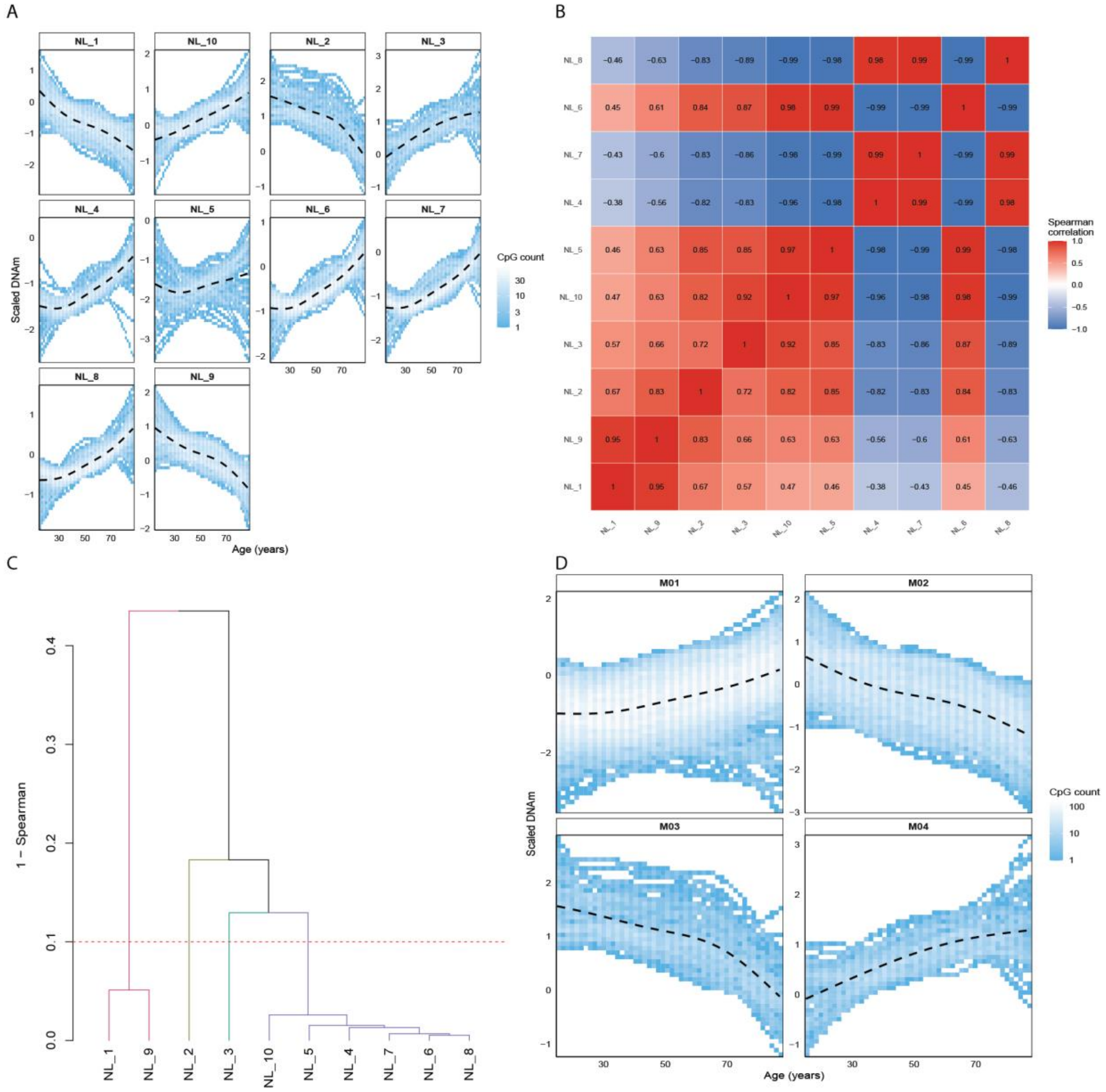

Fig. S10: **A**, Non-linear clusters identified in males after the SNITCH pipeline in Cohort 2. **B,** Correlation matrix between these NL clusters' eigenvalues. **C,** Dendrogram representing the similarity cut-off for merging those clusters. **D,** Resulting cluster after merging in males. Beta values were centered and scaled prior to FPCA and unsupervised.

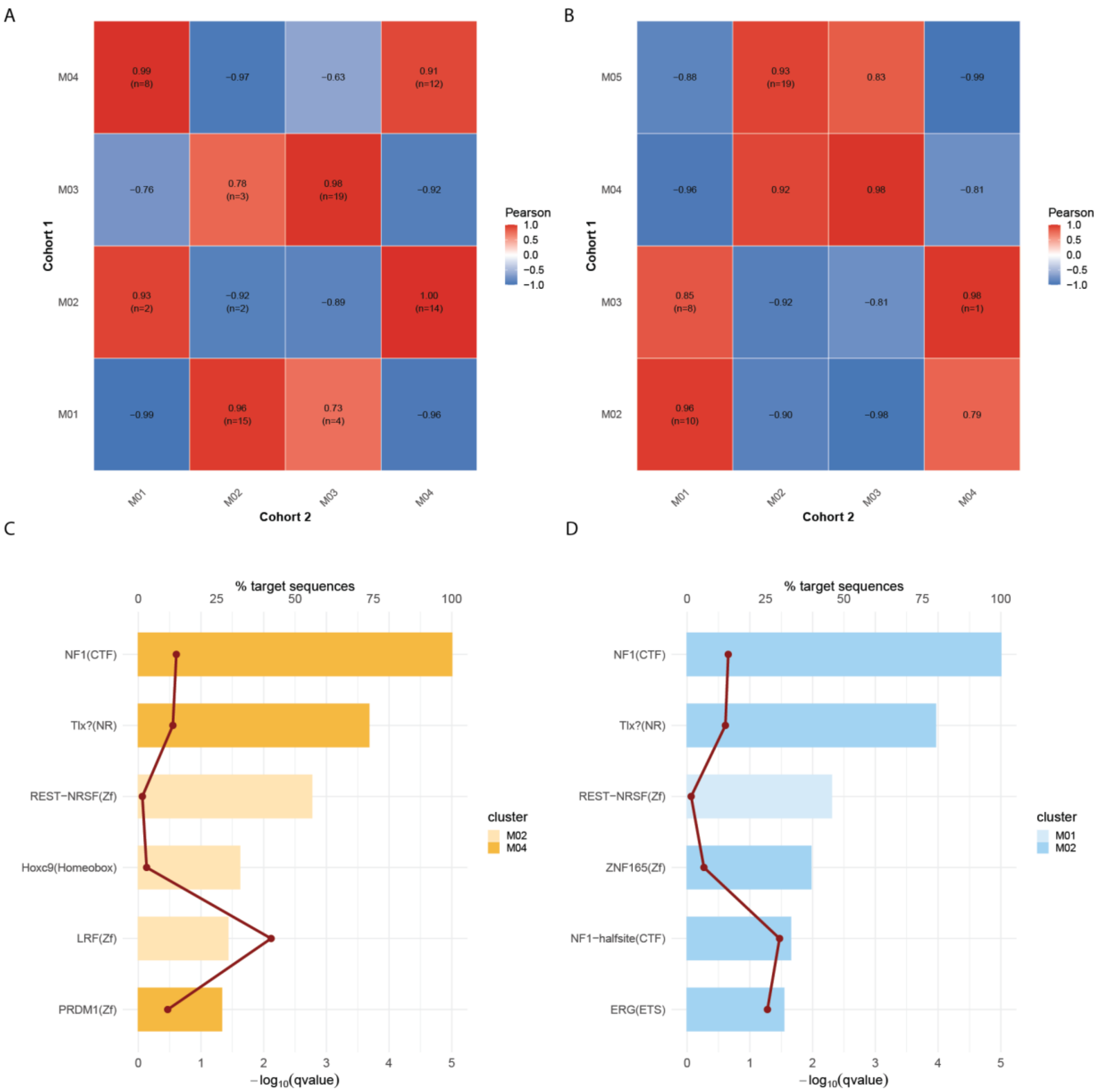

Fig. S11: **A**, Similarity between female clusters across cohorts. The number of overlapping CpGs per cluster is written in each tile. **B,** Similarity between male clusters across cohorts. The number of overlapping CpGs per cluster is written in each tile. **C,** Cluster-wise motif enrichment analysis among females NL CpGs in Cohort 2. **D,** Cluster-wise motif enrichment analysis among males NL CpGs in Cohort 2.

A

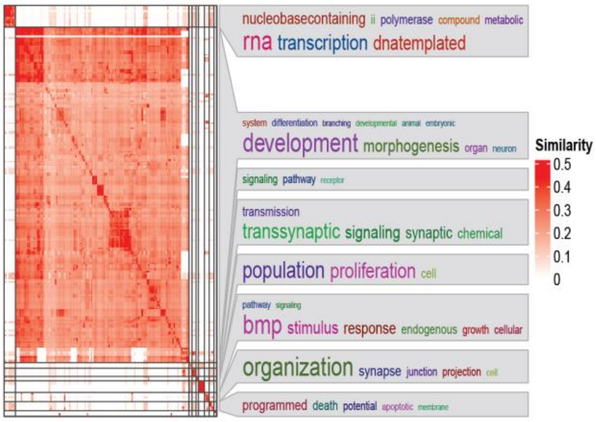

B

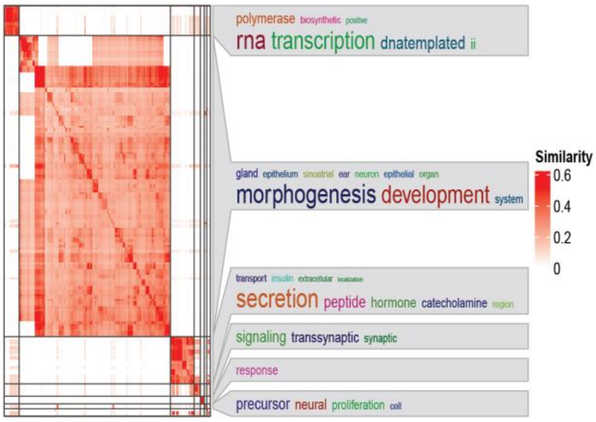

Fig. S12: **A,** Similarity matrix between the terms enriched in the CpGs at the three peaks identified in females M02 of Cohort 2. **B,** Similarity matrix between the terms enriched in the CpGs at the three peaks identified in males M01 of Cohort 2. These were generated using the *simplifyEnrichment* package.

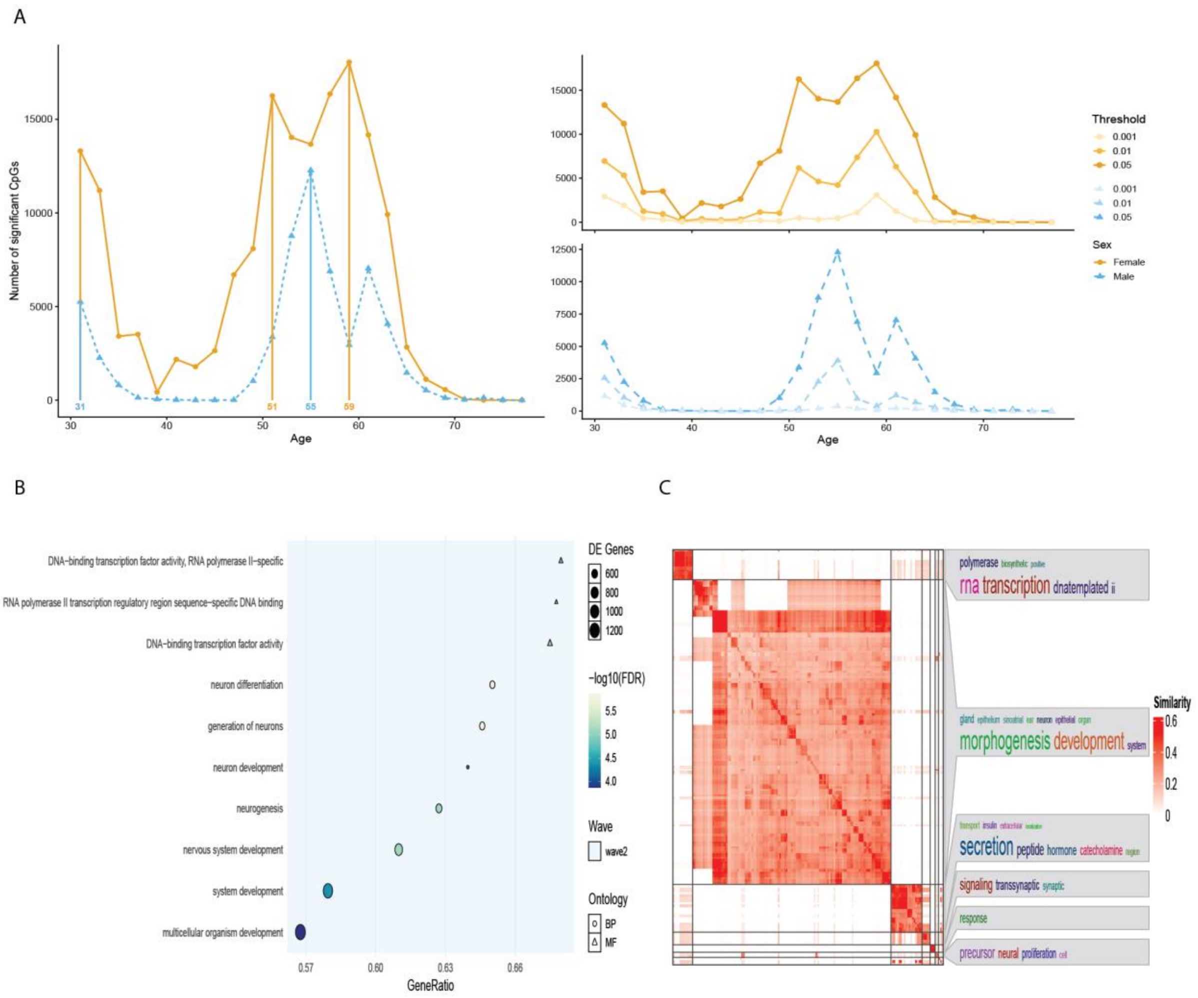

Fig. S13: **A,** Left: DEswan analysis in males and females. Each point represents the number of CpGs dysregulated between a 15-year windows on each side of the specified age at FDR < 0.05. Right: DEswan analysis at different FDR thresholds. **B,** Pathway enrichment analysis on the CpGs at the three peaks identified in males. Only wave 2 shows an enrichment. Top 10 pathways are showed. **C,** Similarity matrix between the terms enriched in the CpGs identified in males wave2 of Cohort 2. These were generated using the *simplifyEnrichment* package.
